# A Novel Non-Resonant Energy Harvester for Ultra-Low-Frequency Energy Harvesting from Human Walking

**DOI:** 10.3390/s26051466

**Published:** 2026-02-26

**Authors:** Guangxian Dong, Yanxi Yu, Weixin Wu, Zhentao Zhang, Yangzi Zhou, Xin Yi, Hongchuan Zhang, Licheng Deng

**Affiliations:** 1 Chongqing Academy of Metrology and Quality Inspection, Chongqing 401121, China; 2 College of Integrated Circuit Science and Engineering, Nanjing University of Posts and Telecommunications, Nanjing 210023, China; dlc@njupt.edu.cn

**Keywords:** energy harvesting, human walking, non-resonant energy harvester, wearable electronics, smart shoe

## Abstract

Harvesting energy from human walking offers a promising alternative to batteries for powering wearable devices. However, existing energy harvesters suffer from limited power output. So, a novel non-resonant energy harvester was proposed in this paper. The core mechanism of the harvester integrates three components: a helical twin-rod twist rod, a face gear with dumbbell-shaped holes, and a rotor featuring bevel teeth on its upper surface. This core mechanism can efficiently harvest low-frequency reciprocating motion and convert it into unidirectional rotational motion, thereby enabling highly efficient acquisition of low-frequency energy. It offers advantages such as high energy harvesting efficiency and a simple structure. Then an electromagnetic generator converts this rotational energy into electricity. A prototype of the proposed harvester was developed and tested on a vertical reciprocating motion platform. Experiments investigated the influence of parameters including human input energy and mechanical harvesting structure on output performance of the harvester. Application testing demonstrated that manual pressing at 1 Hz successfully illuminated 120 LEDs. When integrated into a shoe heel and tested with a 60 kg person stepping in place at 2 steps per second, the harvester achieved an RMS output voltage of 18.5 V, an output power of 263.27 mW, and a power density of 4.21 mW/cm^3^. Overall, this research presents a new approach for designing high-efficiency energy harvesters for human walking applications.

## 1. Introduction

In recent years, the rapid development and widespread application of intelligent wearable electronics devices [1,2] have been significantly propelled by advances in wireless sensor networks [3], the Internet of Things (IoT) [4], and micro-electromechanical systems (MEMS) [5,6]. Intelligent wearable devices have expanded from initial watches and wristbands to encompass broader domains such as smart shoes and smart clothing. Since their market introduction by companies like Adidas and Nike in 2012, smart shoe products have become a prominent branch within the field of intelligent wearable devices [7]. However, the limited battery capacity necessitates frequent charging, causing user inconvenience, and battery disposal poses environmental challenges. Harvesting biomechanical energy from daily human motion and converting it into electricity to power wearable devices presents an effective technical alternative to batteries [8,9]. The various forms of kinetic energy generated by human movement, such as arm swinging [10], knee bending [11], and walking [12], constitute a reliable energy source. Starner et al. [13] investigated the energy potential from a 68 kg person during daily activities including body heat, breathing, blood pressure, typing, arm motion, pedaling, and walking. They found that the foot offers the highest energy potential, at approximately 67 W. Therefore, utilizing energy generated by foot motion to power wearable devices is a viable solution.

Within the past few decades, extensive research has been conducted on harvesting energy from foot motion during human walking. Based on how the excitation from foot motion acts upon the energy harvester, these approaches can be classified into two main categories: direct loading type and inertial excitation type. Direct Loading Type: This refers to directly applying the displacement or force generated by foot motion onto the energy harvester to produce electricity. Xu et al. [14] designed and fabricated an electromagnetic energy harvester based on electromagnetic induction principles to directly collect vertical displacement excitation at the heel. When a 70 kg person walked at 4 steps per second, the harvester generated a voltage of 4.711 V and an energy output of 898.9 μJ per step. Tao et al. [15] developed a triboelectric nanogenerator (TENG) based on a honeycomb structure operating in vertical contact-separation mode, placed in the shoe sole to harvest energy from vertical displacement excitation. The device achieved a maximum instantaneous open circuit voltage of 1207 V, a short-circuit current of 68.5 μA, and a maximum instantaneous power of 12.4 mW. Due to the limited vertical displacement of the foot, energy harvesters based on this scheme often exhibit low output performance. Consequently, researchers have explored harvesters that first convert vertical displacement into rotational motion, which is then converted into electrical energy, enabling higher energy capture. Yin et al. [16] designed a shoe-mounted energy harvester with a cantilever beam structure. This structure converts the vertical displacement of the heel into unidirectional rotation of gears, driving piezoelectric vibration to generate electricity. Experiments showed that at a walking frequency of 4 Hz, the maximum instantaneous power and average power during the upward motion phase were 13.88 mW and 0.98 mW, respectively; during the downward phase, they were 8 mW and 0.18 mW. Research indicates that the maximum impact force during heel strike can reach 1.2 times body weight [17], and this force can act directly on the energy harvester to generate electricity. Qian et al. [18,19] designed a force amplification structure that magnifies vertical pressure before transferring it to a piezoelectric stack to maximize output power. Experimental results demonstrated that when an 84 kg person walked at 5.6 km/h, the average power reached 20 mW and the maximum instantaneous power reached 204.7 mW. Asano et al. [20] also designed a foot energy harvester with a parallel linkage structure featuring force amplification. Experimental results indicated that when a person of average weight walked at a step frequency of 1 Hz, the average power reached 1.29 mW and the maximum instantaneous power reached 20.3 mW. During human walking, the sole undergoes periodic bending motion synchronized with the gait cycle, which can be utilized as an energy source. Wang et al. [21] designed an electromagnetic energy harvester based on sole bending. This device converts the bending motion of the sole into unidirectional rotation of the generator shaft. Experimental results showed that when a 72 kg person walked at 7 km/h, the harvester achieved an average power of approximately 10 mW. Inertial Excitation Type: During human walking, the foot typically undergoes periodic vertical motion. This motion can be regarded as periodic vibration and serves as inertial excitation applied to the energy harvester, converting human motion energy into electricity. Wang et al. [22] designed an electromagnetic vibration energy harvester based on electromagnetic induction principles to collect vibration excitation from the foot during walking. Experimental results showed that the converted electrical power reached up to 7.71 mW when a person walked at 8 km/h. However, constrained by the low frequency of human motion-induced vibrations, this structure suffers from low average output power.

The aforementioned studies indicate that despite extensive research in the field of shoe-based energy harvesters, certain limitations persist, including low output power and structural complexity. To address these issues, this paper proposes a non-resonant energy harvester designed to harvest ultra-low-frequency energy generated during human walking. The core structure of this harvester consists of three key components working in synergy: a twist bar with a double-helix structure, a face gear with dumbbell-shaped holes, and a rotor featuring inclined teeth on its upper surface. This design efficiently harvests energy from low-frequency reciprocating motion and converts it into unidirectional rotational energy, thereby enabling the highly efficient acquisition of low-frequency energy. It offers advantages such as high energy-harvesting efficiency and a simple structure. The remainder of this paper is organized as follows: Section 2 presents the novel structure of the energy harvester and explains its working principle. Section 3 details the fabrication of the harvester prototype, the setup of the testing platform, and experimental investigations into the influence of key structural parameters on output performance. Section 4 reports on application testing of the developed harvester. Finally, Section 5 provides the conclusions.

## 2. Device Design and Working Mechanism Analysis

**Figure 1:**
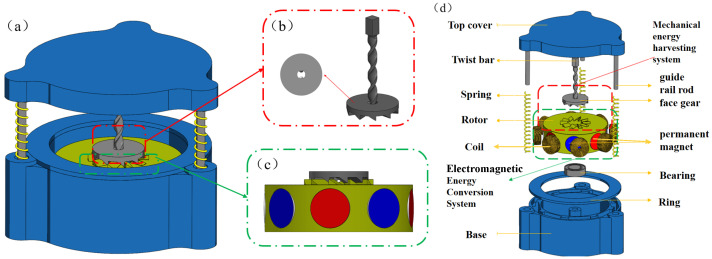
The schematic of the proposed NR-EH. (**a**) Overall view, (**b**) The detail view of the face gear and the twist bar, (**c**) The detail view of the rotor, (**d**) Exploded view.

Figure 1 illustrates the novel non-resonant energy harvester (NR-EH) structure proposed in this paper. The NR-EH primarily consists of two subsystems: a mechanical energy harvesting system and a electromagnetic energy conversion system. The mechanical energy harvesting system comprises a double-helix structured twist bar, a face gear with beveled teeth and entrally-located dumbbell-shaped aperture fit together with the twist bar, and a rotor with bevel teeth on the upper surface fit together with the face gear. The electromagnetic energy harvesting system is mainly composed of permanent magnets and coils. The permanent magnets are installed on the rotor, and the coils are installed in the base. Different colors of the permanent magnets indicate different magnetic pole directions. The rotor is mounted on the base via bearing. The twist bar is rigidly mounted to the top cover while being capable of reciprocating axially through the central bores of the face gear and rotor. To restrict the twist bar to reciprocating motion solely along its longitudinal axis, three spring-loaded guide rods have been installed between the top cover and the base.

**Figure 2:**
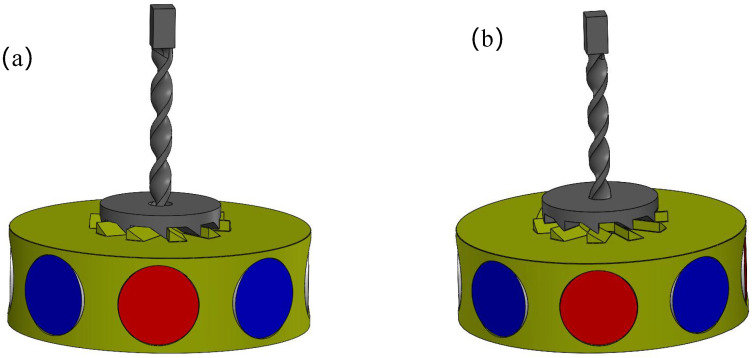
The contact relationship between the face gear and the gear of the rotor under different motion states of the twist bar (**a**) The twist bar downwards, (**b**) The twist bar either remains stationary or moves upward.

When vertical impact or compression is applied on the device, the top cover of the NR-EH can drive the twist bar downwards. At this time, the face gear will be subjected to a downward force and a horizontal force, the downward force causes the face gear to mesh with the gear of the rotor, as shown in Figure 2a, and the horizontal force drives the face gear to rotate, thus driving the rotor to rotate together. The magnets installed on the rotor rotate synchronously with it, thereby changing the magnetic flux in the coil fixed to the base, which generates an induced current in the coil. When the external force on the NR-EH is maintained or removed, the twist bar either remains stationary or moves upward, and the face gear either stays motionless or rotates in the opposite direction. At this stage, contact transitions from tooth meshing to direct interaction between the inclined flanks of the face gear teeth and rotor teeth, as shown in Figure 2b. The face gear no longer provides a driving force to the rotor and the rotor continues to rotating by inertia with minimal friction. In summary, this structure enables both efficient low-frequency energy harvesting and the conversion of reciprocating motion into unidirectional rotation. Compared with the use of a unidirectional ratchet mechanism to convert reciprocating motion into unidirectional rotation [23], the proposed structure is simpler. It is highly suitable for harvesting the energy from the ultra-low-frequency motion of human walking.

## 3. Prototype and Experimental Setup

**Figure 3:**
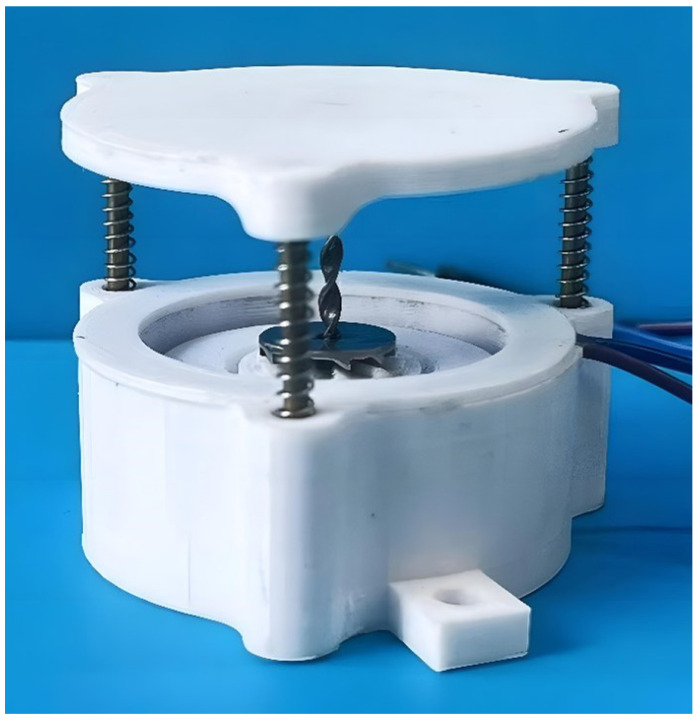
The fabriated NR-EH.

**Figure 4:**
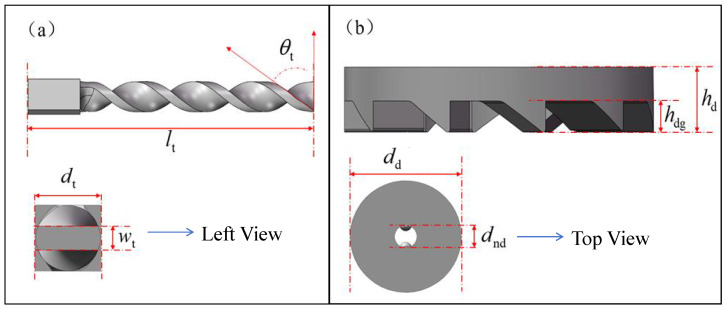
The structural parameters of NR-EH components (**a**) Twist bar, (**b**) Face gear.

**Figure 5:**
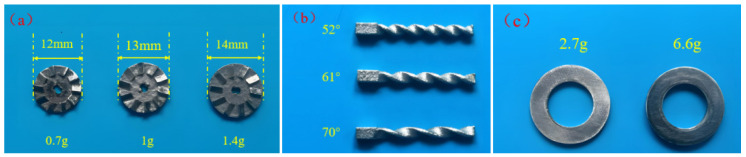
The fabricated NR-EH components with varied structural parameters (**a**) Face gear, (**b**) Twist bar, (**c**) Counterweight ring.

Figure 3 shows the fabricated NR-EH prototype. The structural parameters are listed in the Table 1. Figure 4 shows structural parameters of the twist bar and face gear. In Table 1, $d_{r}$, $h_{r}$ and $h_{rg}$ represent the diameter, thickness and tooth thickness of the rotor, respectively. The base, top cover, ring structure, and rotor (excluding the magnets) of the prototype were manufactured using 3D printing technology with PLA material. The twist bar and face gear were also 3D-printed using stainless steel, as illustrated in Figure 5a,b. The permanent magnet, coil, bearing, guide rail rod and the spring on it are all general products, which were directly purchased.

Based on the working principle and structural design of the NR-EH device, the parameters affecting its performance can be categorized into three main types: input energy (including the compression frequency and compression amplitude of the device); mechanical energy harvesting structure (mainly comprising the helix angle of the twist bar, the mass of the face gear, and the mass of the rotor); and electromechanical energy conversion structure. Since this paper adopts a permanent magnet coil structure for electromechanical energy conversion, which has been relatively well-researched, detailed analysis of this part will not be conducted in this paper.

**Figure 6:**
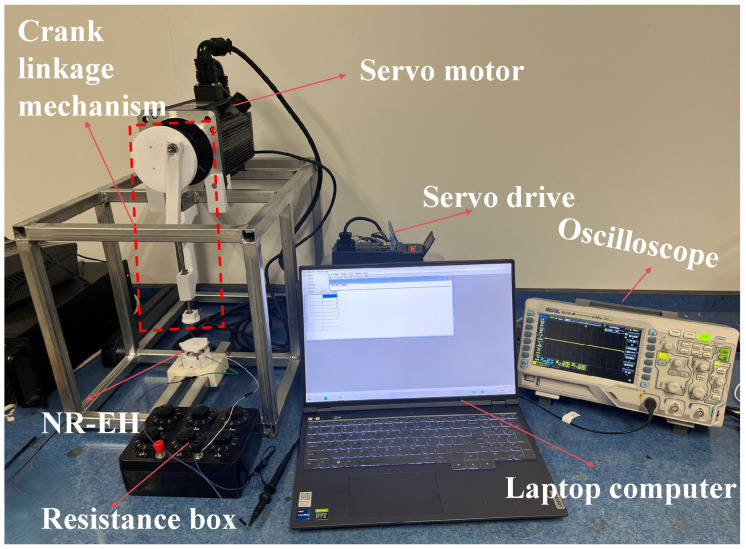
The experimental test system.

During human walking, the heel experiences significant motion amplitude and pressure. Therefore, the NR-EH is installed in the heel area of footwear to harvest energy generated during walking. To simulate the reciprocating motion of the heel against the shoe during human walking, an experimental test system (shown in Figure 6) was constructed to evaluate the device’s performance. In order to investigate the effects of face gear mass, twist bar helix angle, and rotor mass on the output performance of the NR-EH, three face gears with different diameters were fabricated (Figure 5a) with masses of 0.7 g, 1 g, and 1.4 g, respectively, three twist bars with helix angles of 52°, 61°, and 70° were also manufactured (Figure 5b), and two counterweight rings with masses of 2.7 g and 6.6 g were produced (Figure 5c) to increase the rotor mass. To quantify the output power or electrical energy, a resistor is employed as the device’s load in this study.

This experimental test system primarily consists of the following key components: a servo motor, a crank linkage mechanism, and an oscilloscope. The servo motor (model: POWSM-T-H2-130-15025-AS4B62, Zhejiang Guomai Technology Co., Ltd., Wenzhou, Zhejiang Province, China), controlled by a servo driver (model: SUPSD-U2P-50APB, Zhejiang Guomai Technology Co., Ltd., Wenzhou, Zhejiang Province, China) generates rotation at adjustable speeds to drive the crank linkage mechanism. This mechanism produces reciprocating motion that mimics the heel’s movement during walking, as illustrated in Figure 6. The fabricated NR-EH is mounted beneath the crank linkage mechanism and undergoes continuous reciprocating compression. An oscilloscope (Rigol DS1102Z-E, Rigol Technologies Co., Ltd., Suzhou City, Jiangsu Province) is employed to sample and analyze the device’s output signals.

## 4. Experimental Results and Discussion

### 4.1. The Optimal Load Resistance

The load resistance influences the output performance of the device. To accurately characterize the device’s output performance, this study first experimentally determines the optimal load resistance. Tests were conducted on a prototype with typical structural parameter values under rotational excitation at 90 RPM and a compression amplitude of 15 mm.

**Figure 7:**
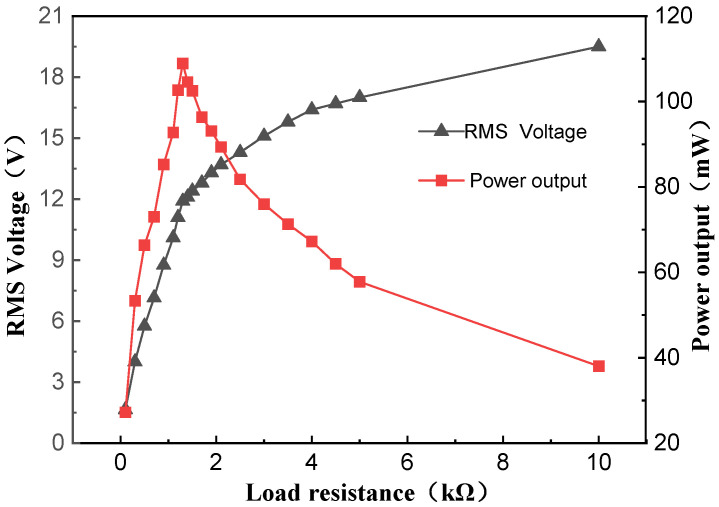
The effect of load resistance on NR-EH output characteristics.

To investigate the output performance of the device, load resistors were connected with the device. Experimental results are shown in Figure 7. As illustrated, the RMS voltage across the load resistor increases with increasing load resistance, while the output power reaches its peak value at 1.3 kΩ. This specific load resistance corresponds to the optimal load resistance for the NR-EH system.

### 4.2. Effect of Input Energy on Output Performance of NR-EH

During the human walking process, energy is input into the NR-EH, causing it to generate electrical energy output. Human walking can be characterized by two parameters: compression frequency and compression amplitude. In the experiment, the compression frequency and amplitude were adjusted by changing the rotational speed of the servo motor and the length of the crank-link mechanism. The following analyzes the influence of compression frequency and amplitude on the output performance of NR-EH respectively.

**Figure 8:**
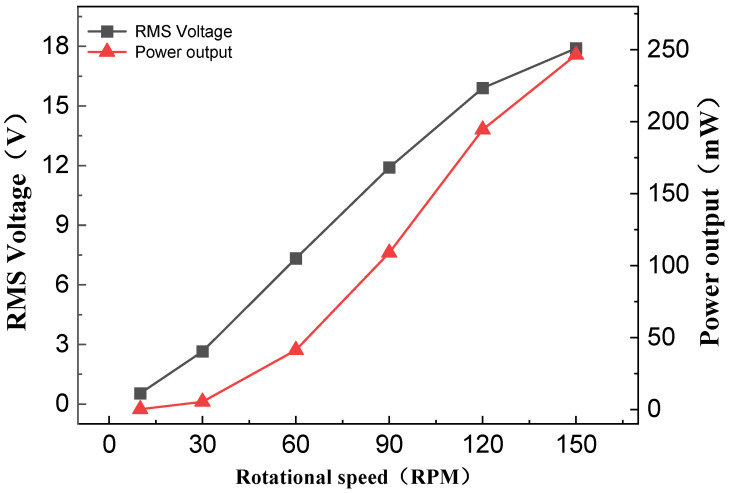
The effect of rotational speed on NR-EH output characteristics.

Figure 8 shows the test curves of the effect of compression frequency on the output performance of NR-EH. The test was conducted using a principle prototype with typical structural parameter values. The compression amplitude was set to 15 mm, the load resistance was 1.3 kΩ, and the compression frequency was adjusted by changing the rotational speed of the servo motor. The rotational speed ranged from 0 to 150 RPM, corresponding to a frequency range of 0–2.5 Hz. It can be seen that the RMS voltage and output power increase with the increase in compression frequency. Before 120 RPM, the increase in RMS voltage is approximately linear, while beyond 120 RPM, the growth curve droops. The main reason is that as the rotational speed increases, the torque provided by the servo motor decreases. The decrease in torque leads to a reduction in the transmitted compression force, which makes the reaction force of the rotor more prominent and has a negative impact on the output.

The compression amplitude significantly impacts not only the structural size of the NR-EH but also its output performance. Therefore, it is essential to conduct an in-depth study on the influence of compression amplitude on the NR-EH’s output performance. In the experiment, only the compression amplitude was varied (achieved precisely using modules of different fixed heights to obtain specific amplitude values), while all other structural parameters employed the typical values of the prototype. The rotational excitation was fixed at 90 RPM.

**Figure 9:**
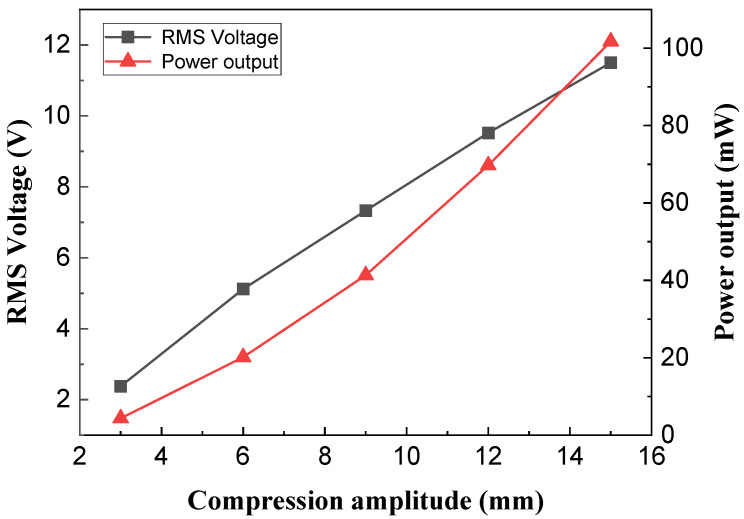
The effect of compression amplitude on NR-EH output characteristics.

Figure 9 shows the test curves depicting the relationship between compression amplitude and output performance. It is evident that the compression amplitude has a significant impact on the output performance of the NR-EH. As the compression amplitude increases, the output RMS voltage correspondingly increases, exhibiting an approximately linear growth trend. Concurrently, the output power also increases with higher compression amplitudes.

It can be seen that the compression amplitude has a significant influence on the NR-EH’s output performance. To balance structural compactness with output performance, a compression amplitude of 15 mm was uniformly adopted in subsequent testing.

### 4.3. Effect of Mechanical Energy Harvesting Structure on Output Performance of NR-EH

The primary mechanical energy harvesting structure comprises a face gear, a rotor, and a twist bar. The mass of the face gear, the mass of the rotor, and the helical angle of the twist bar all influence the output performance of the NR-EH. Experimental investigations for each parameter are conducted separately below.

**Figure 10:**
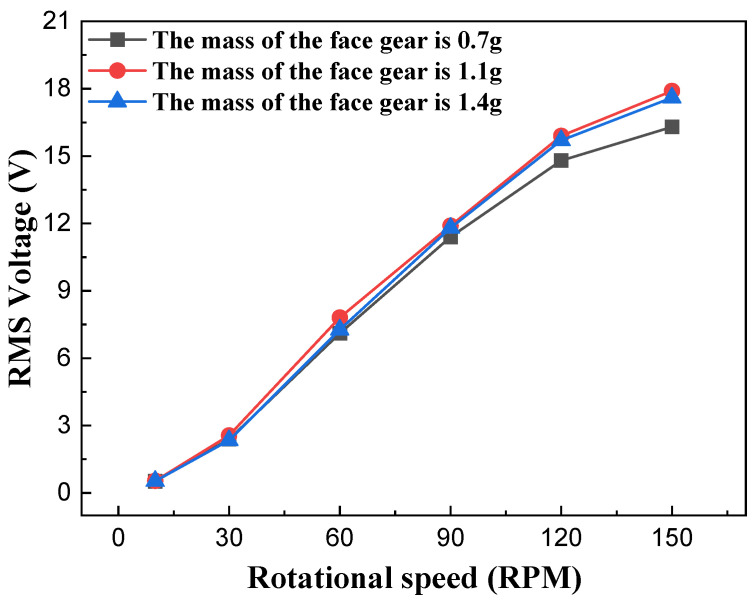
The effect of the face gear mass on the voltage of the NR-EH.

**Figure 11:**
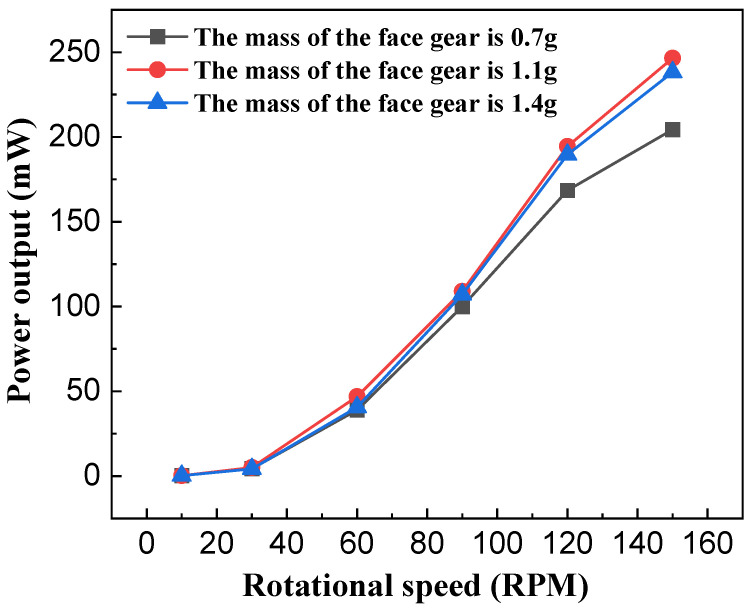
The effect of the face gear mass on the power output of the NR-EH.

To investigate the effect of the face gear’s mass on the output performance of the NR-EH, only the diameter of the face gear (representing the face gear mass) was varied in the experiments, while all other structural parameters employed the typical values from the prototype. Testing was performed within a rotational excitation range of 0 to 150 RPM. Figure 10 and Figure 11 show the test curves depicting the relationship between face gear mass and output performance under compressive excitation. It can be observed that when the face gear mass is 1g, the RMS output voltage and output power of the NR-EH are relatively superior. Both excessively light and excessively heavy masses of the small disc lead to a reduction in output performance. This phenomenon can be explained as follows: During the compression process, the face gear descends to engage with the teeth on the rotor. A faster descent allows the energy on the face gear to be transferred to the rotor more rapidly. The descent speed of the face gear is influenced by its own gravitational force and the friction with the twist bar. When its own mass is too small, the descent is slow, preventing the rapid transfer of energy from the face gear to the rotor. Conversely, during the compression unloading process, the rotor continues to rotate due to inertia. At this point, the face gear remains in contact with the rotor under its own gravitational force, thus generating a friction force, and impeding the rotor’s motion. A larger face gear mass results in stronger hindrance. Therefore, the mass of the face gear should neither be too large nor too small. To ensure optimal output performance of the NR-EH across various rotational excitations, a face gear mass of 1g was uniformly adopted in all subsequent tests.

**Figure 12:**
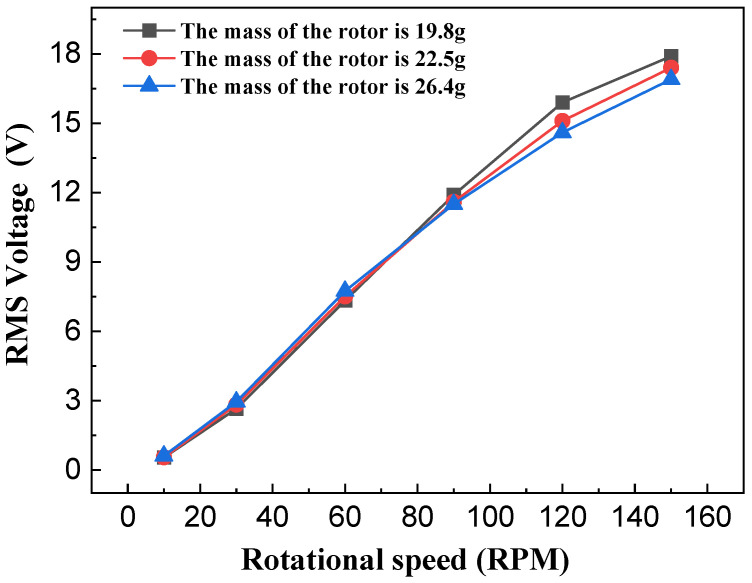
The effect of the rotor mass on the voltage of the NR-EH.

**Figure 13:**
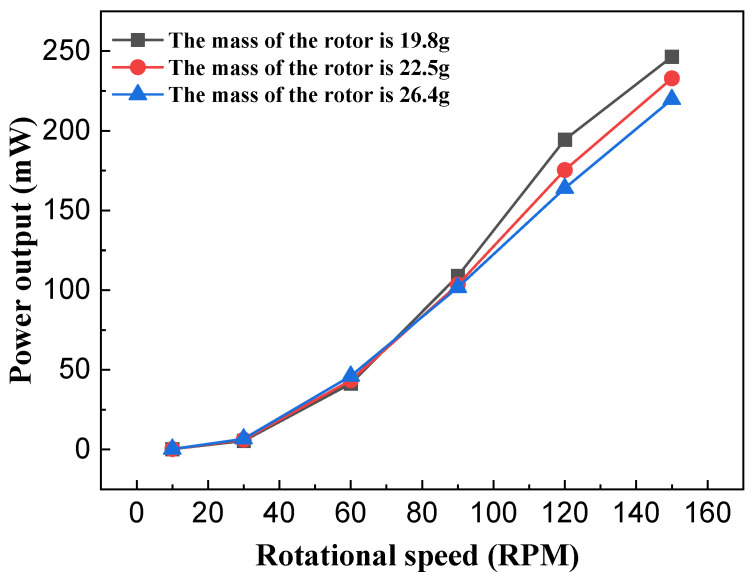
The effect of the rotor mass on the power output of the NR-EH.

According to the kinetic energy formula, at a constant rotational speed, a larger rotor mass results in greater kinetic energy acquired. This also indicates that rotor mass significantly influences the output performance of the NR-EH. In the experiments, only the rotor mass was varied, while other parameters maintained the structural values typical of the prototype. The NR-EH was experimentally tested under rotational excitation ranging from 0 to 150 RPM. Figure 12 and Figure 13 show the test curves depicting the relationship between rotor mass and output performance of the NR-EH. It can be observed that prior to the excitation reaching 75 RPM, larger rotor masses yielded better output performance, which aligns with theoretical expectations. However, when the rotational excitation exceeded 75 RPM, the output performance degraded with increasing rotor mass. This phenomenon occurs because the servo motor provides a reduced compressive force at higher rotational angular velocities. This diminished force allows the reaction force of the rotor to become more pronounced, consequently impairing the output performance of the NR-EH. In subsequent tests, to minimize the impact of rotor reaction forces on the experiments, a rotor mass of 19.8 g was consistently employed for all experimental analyses.

The twist bar plays a crucial role in transmitting compressive energy within the NR-EH, and its helix angle is a key parameter influencing energy transfer. Geometric analysis reveals the relationship: $\omega_{t}=\frac{2V_{t}}{d_{t}sin\theta_{t}}$, where $\theta_{t}$ represents the helix angle of the twist bar, $V_{z}$ the vertical compression velocity, and $d_{t}$ the diameter of the twist bar. It can be deduced that a smaller helix angle results in a higher transmitted rotational angular velocity. To gain a deeper understanding of this relationship, experiments were conducted where only the helix angle of the twist bar was varied, while the initial parameter settings of other critical components remained unchanged. Tests were performed under continuous rotational excitation ranging from 0 to 150 RPM.

**Figure 14:**
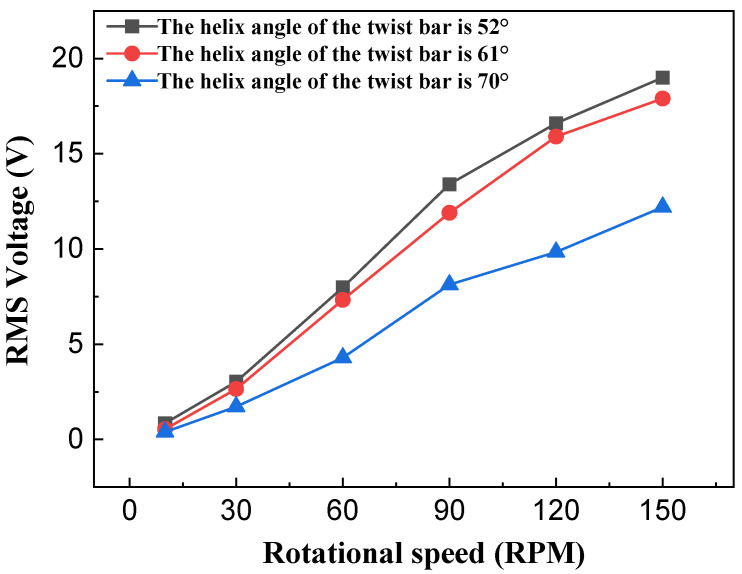
The effect of the helix angle of the twist bar on the voltage of the NR-EH.

**Figure 15:**
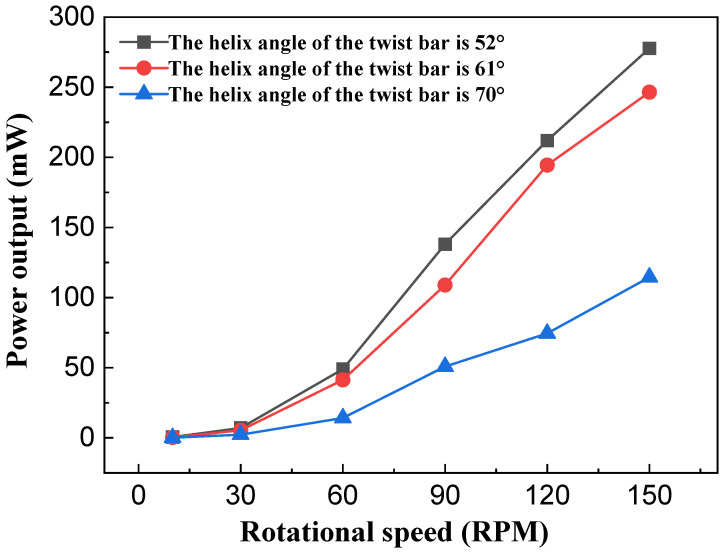
The effect of the helix angle of the twist bar on the power output of the NR-EH.

Figure 14 and Figure 15 show the test curves depicting the relationship between the helix angle of the twist bar and output performance of the NR-EH. It can be observed that output performance decreases as the helix angle increases, which aligns with the theoretical analysis. However, as the helix angle decreases, the rate of improvement in output performance diminishes. The underlying reasons are twofold: (1) A smaller helix angle enables the acquisition of greater energy during the compression phase, leading to higher energy transfer to the rotor; (2) after compression ceases, the rotor continues to rotate, generating friction between the face gear and the rotor which impedes its motion. This frictional resistance increases as the helix angle decreases. The combined effect of these two factors leads to the observed slowing rate of output performance enhancement. Therefore, a smaller twist bar helix angle is not always better.

## 5. Applications

**Figure 16:**
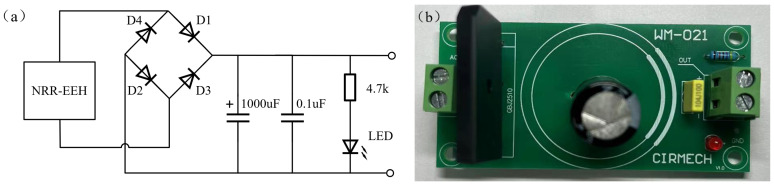
The (**a**) schematic diagram and (**b**) physical implementation of the capacitor charging circuit.

**Figure 17:**
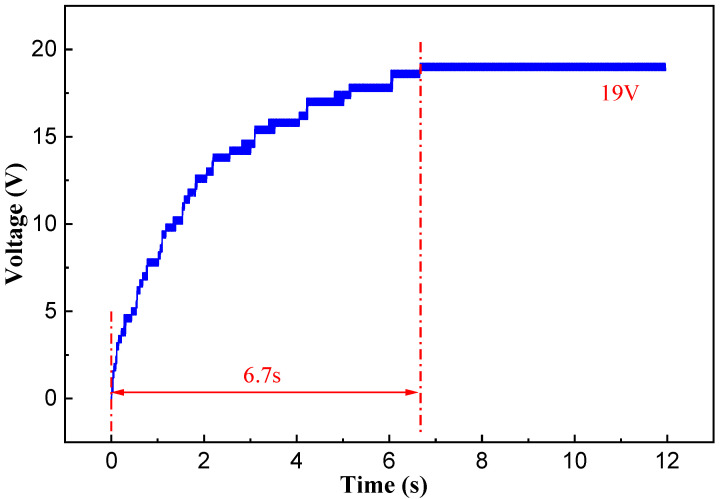
The charging curve of the NR-EH.

**Figure 18:**
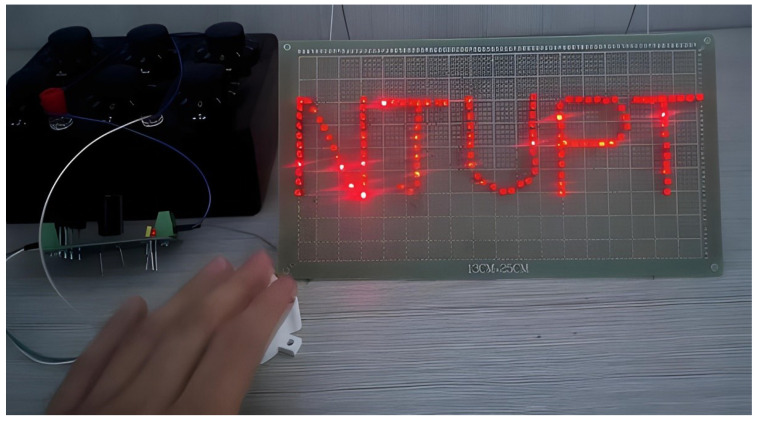
Manual compression test diagram of the NR-EH.

Following the investigation of the output performance of the developed NR-EH, our research shifted to examining its practical application in real-world environments. The first phase involved characterizing the harvester’s capacitive charging behavior. The developed NR-EH was connected to a bridge rectifier circuit to charge a capacitor. Figure 16 shows the schematic diagram and physical implementation of the capacitor charging circuit. Under a rotational excitation of 120 RPM, the charging curve is presented in Figure 17. Experimental results demonstrate that a 1000 μF capacitor was successfully charged to 19 V within 6.7 s. Furthermore, via manual pressing at a frequency of once per second, the NR-EH successfully illuminated a display panel consisting of approximately 120 LEDs (each with a threshold voltage of 2 V and power consumption of 0.1 mW), as shown in Figure 18.

**Figure 19:**
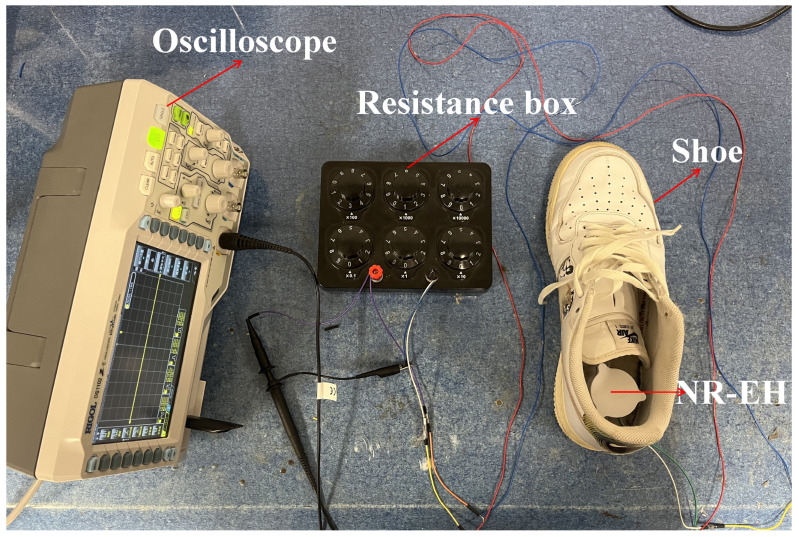
Schematic diagram of the heel-mounted NR-EH Test Setup.

Finally, the developed NR-EH was mounted at the shoe heel to harvest energy from human motion. The testing setup is shown in Figure 19. The NR-EH utilized the structural parameter values typical of the prototype, specifically: a twist bar with a 61°helix angle, a face gear mass of 1 g, a rotor mass of 19.8 g, and a compression amplitude of 15 mm. The overall height of the prototype was 39 mm.

**Figure 20:**
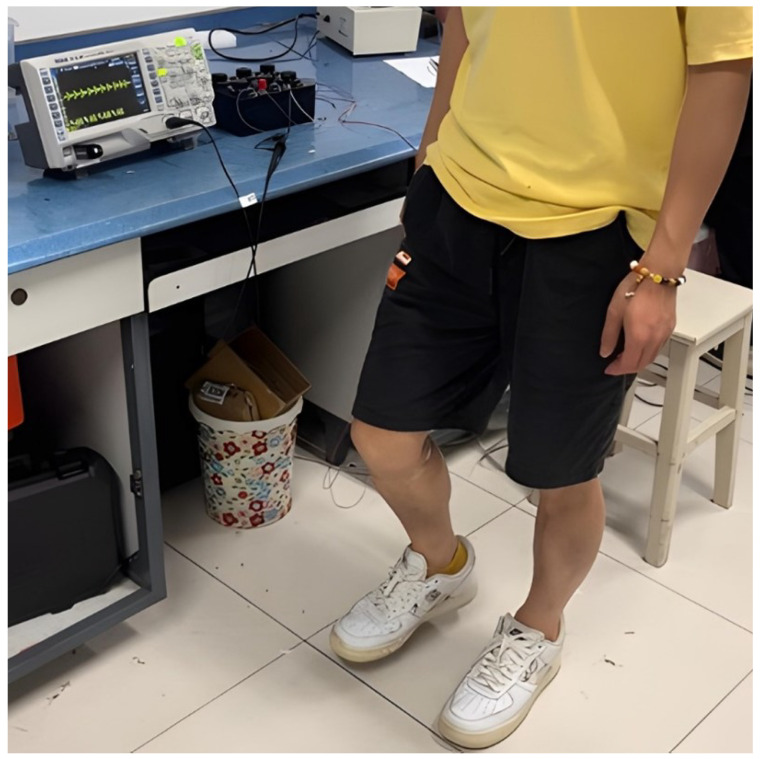
Field-testing scenario of the NR-EH during human walking trials.

**Figure 21:**
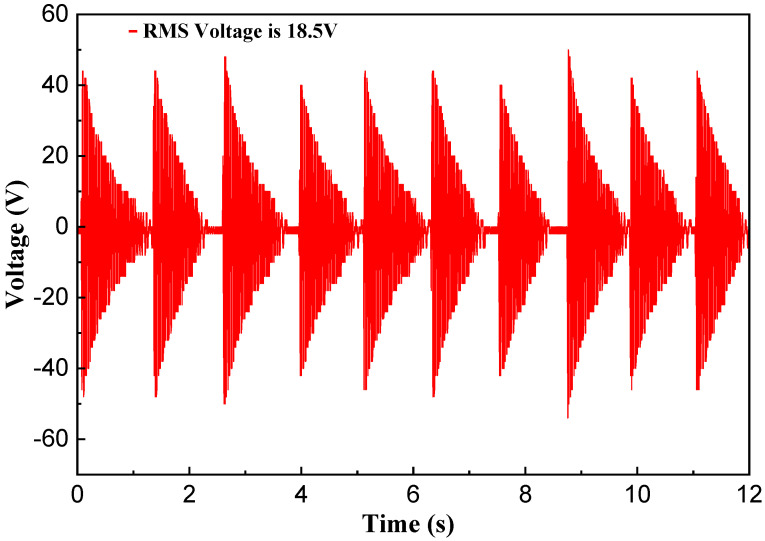
The load voltage of the NR-EH during human walking.

**Figure 22:**
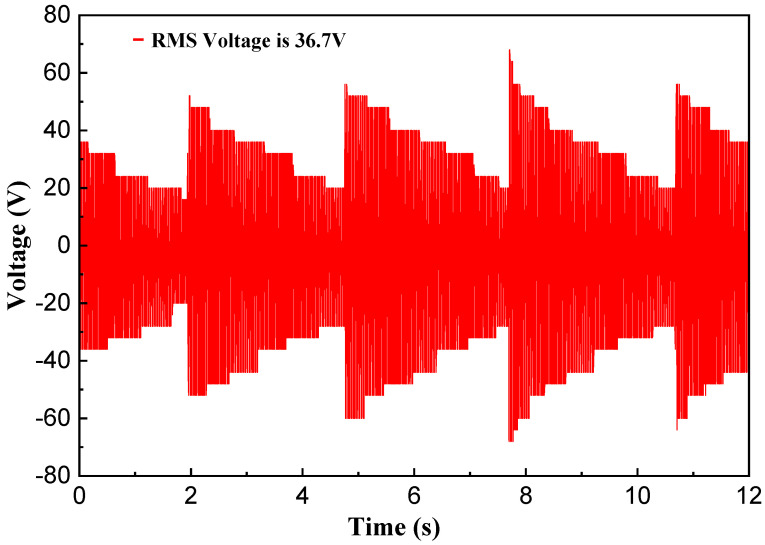
The open circuit voltage of the NR-EH during human walking.

During the experiment, a volunteer weighing 60 kg wore the shoes equipped with the NR-EH and performed stationary stepping at a cadence of 2 steps per second, as depicted in Figure 20. The resulting output voltage waveform is shown in Figure 21. Under test conditions with an external load resistance of 1.3k Ω, the RMS voltage was measured at 18.5 V, yielding a calculated output power of 263.27 mW. Based on estimation, approximately 315.9 mJ of energy was harvested per step. Given the NR-EH volume of 62.5 cm^3^, the corresponding power volumetric density was 4.21 mW/cm^3^. Subsequently, the open-circuit output voltage (without any load resistor) was measured at the same stepping frequency, as presented in Figure 22, resulting in an RMS voltage of 36.7 V. The output performance of the prototype was further tested under conditions of stationary stepping by volunteers at different cadences, with the corresponding results presented in Table 2. It can be seen that the trends in the test results demonstrate a high degree of consistency with those obtained from the prototype on the experimental test platform. Table 3 presents a comparison between the proposed design and previously reported shoe-mounted energy harvesters. Based on the comparative data, the energy harvester developed in this work demonstrates superior performance in both power output and power density, exhibiting greater potential for practical applications.

## 6. Conclusions

To efficiently harvest ultralow-frequency kinetic energy from human walking, this paper proposes a novel non-resonant kinetic energy harvester. The core structure of this harvester is a mechanical energy harvesting mechanism organically integrating three components: a twist rod with a helical twin-rod structure, a face gear with dumbbell-shaped holes, and a rotor with bevel teeth on its upper surface. The synergistic interaction among these three structures enables the dual functionalities of low-frequency energy harvesting and a unidirectional ratchet mechanism. Thereby, the system not only achieves high-efficiency energy acquisition but also exhibits inherent structural simplicity. So, the proposed harvester can effectively convert the vertical reciprocating motion during human walking into unidirectional horizontal rotation of the rotor, thereby enabling efficient capture of human motion energy. Subsequently, an electromagnetic structure converts the captured rotational energy of the rotor into electrical energy. Following this, a prototype of the non-resonant kinetic energy harvester was developed. A vertical reciprocating motion test platform was established to experimentally investigate the influence of parameters such as human input energy and the mechanical energy harvesting structure on the output performance of the harvester. Finally, application experiments were conducted. Manual pressing of the developed prototype at a frequency of 1 Hz successfully illuminated 120 LED lights. When the prototype was installed in the heel of a shoe and tested with a 60 kg person stepping in place at a cadence of 2 steps per second, the harvester achieved a root mean square (RMS) output voltage of 18.5 V, an output power of 263.27 mW, and a power density of 4.21 mW/cm^3^. This research may provide a new way to design high-efficiency energy harvesters used for human walking energy harvesting.

## Figures and Tables

**Table 1:** Parameters of the fabricated NR-EH.

Component	Structural Parameter	Value
Twist bar	$\theta_{t}$(°)	61/52 */70 *
	$l_{t}$; $d_{t}$ (mm)	22; 2
Face gear	$d_{d}$ (mm)	13/12 */14 *
	$h_{d},h_{dg}$ (mm)	2.1; 1.1
	Number of teeth	10
	Mass (g)	1/0.7 */1.4 *
Rotor	$d_{r};h_{r};h_{rg}$ (mm)	15; 11.1; 1.1
	Number of teeth	10
	Mass (g)	19.8/22.5 */26.4 *
Permanent Magnet	Radius; Thickness (mm)	4; 5
	Distance from the Magnet to the Rotor Center (mm)	15
Coil	In/Outer diameter/Thickness (mm)	1; 4.4; 3
	Number of turns	1850
	Distance from the Coil to the Rotor Center (mm)	16.5
The top cover	Cross-sectional area (mm^2^)	1605.12
	Height (mm)	5
Guide Rod	Diameter, Length (mm)	2; 22
Spring	Diameter, Wire Diameter, Length (mm)	3; 0.3; 30
The base	Height (mm)	19
	Cross-sectional area (mm^2^)	1605.12
Bearing	In/Outer diameter; Thickness (mm)	3; 5; 3
	Compression amplitude (mm)	15/12 */9 */6 */3 *

Note: Values with asterisk (*) denote alternative specifications.

**Table 2:** The output performance of the prototype at different cadences.

Cadence (Steps per Second)	Load Voltage (V)	Power Output (mW)	Power Density (mW/cm^3^)
1	9.3	66.53	1.06
2	18.5	263.27	4.21
3	27.2	569.11	9.11

**Table 3:** Performance comparison with reported human walking energy harvesters.

Reference	Excitation	Power Output (mW)	Volume (cm^3^)	Power Density (mW/cm^3^)
[15]	Hand pressing	12.4	30	0.413
[16]	Walking at 4 Hz	0.98	75.25	0.013
[18]	Walking at 5.6 km/h (84 kg person)	20	6.27	3.19
[20]	Walking at 1 Hz	1.29	76.56	0.017
[21]	Walking at 5.6 km/h	10	22.61	0.44
[24]	Walking at 4 km/h	97	82.8	1.17
[23]	Walking at 9 km/h	85	46.2	1.84
**This work**	**Stepping at 2 Hz (60 kg person)**	**263.27**	**62.5**	**4.212**

## Data Availability

The original contributions presented in this study are included in the article. Further inquiries can be directed to the corresponding author.

## References

[1] Liu F., Han J.L., Qi J., Zhang Y., Yu J.L., Li W.P., Lin D., Chen L.X., Li B.W. (2021). Research and Application Progress of Intelligent Wearable Devices. Chin. J. Anal. Chem..

[2] Wang Y., Zou Y., Li Z. (2024). Emerging intelligent wearable devices for cardiovascular health monitoring. Nano Today.

[3] Yu X., Fu Y., Li J., Mao J., Hoang T., Wang H. (2024). Recent advances in wireless sensor networks for structural health monitoring of civil infrastructure. J. Infrastruct. Intell. Resil..

[4] Yalli J.S., Hasan M.H., Jung L.T., Al-Selwi S.M. (2025). Authentication schemes for Internet of Things (IoT) networks: A systematic review and security assessment. Internet Things.

[5] Pillai G., Li S.S. (2021). Piezoelectric MEMS Resonators: A Review. IEEE Sens. J..

[6] Malayappan B., Lakshmi U.P., Rao B.V.V.S.N.P., Ramaswamy K., Pattnaik P.K. (2022). Sensing Techniques and Interrogation Methods in Optical MEMS Accelerometers: A Review. IEEE Sens. J..

[7] Rhoades M.A., McCann J., Bryson D. (2023). 18—Smart footwear: A designer’s perspective. Smart Clothes and Wearable Technology (Second Edition).

[8] Dong Y., Duan L., Mao X., Gu T., Gu Y., Yu H., Zhang X., Ye T., Wang X., Li P. (2025). Exploring human motions for smart wearables: Energy conversion, harvesting and self–powered sensing. Nano Energy.

[9] Liu M., Qian F., Mi J., Zuo L. (2022). Biomechanical energy harvesting for wearable and mobile devices: State-of-the-art and future directions. Appl. Energy.

[10] Cai M., Liao W.H. (2021). Enhanced electromagnetic wrist-worn energy harvester using repulsive magnetic spring. Mech. Syst. Signal Process..

[11] Hao D., Li Y., Wu J., Zeng L., Zhang Z., Chen H., Liu W. (2024). A self-powered and self-sensing knee negative energy harvester. iScience.

[12] Zou J., Huang J., Pei J., Yang X., Huang Z., Liu K. (2024). A walking energy harvesting device based on miniature water turbine. J. Appl. Phys..

[13] Starner T. (1996). Human-powered wearable computing. IBM Syst. J..

[14] Xu B., Li Y. (2019). Force analysis and energy harvesting for innovative multi-functional shoes. Front. Mater..

[15] Tao K., Chen Z., Yi H., Zhang R., Shen Q., Wu J., Tang L., Fan K., Fu Y., Miao J. (2021). Hierarchical honeycomb-structured electret/triboelectric nanogenerator for biomechanical and morphing wing energy harvesting. Nano-Micro Lett..

[16] Yin Z., Gao S., Jin L., Guo S., Wu Q., Li Z. (2021). A shoe-mounted frequency up-converted piezoelectric energy harvester. Sens. Actuators A Phys..

[17] Niu P., Chapman P., Riemer R., Zhang X. (2004). Evaluation of motions and actuation methods for biomechanical energy harvesting. Proceedings of the 2004 IEEE 35th Annual Power Electronics Specialists Conference (IEEE Cat. No.04CH37551), Aachen, Germany, 20-25 June 2004.

[18] Qian F., Xu T.B., Zuo L. (2018). Design, optimization, modeling and testing of a piezoelectric footwear energy harvester. Energy Convers. Manag..

[19] Qian F., Xu T.B., Zuo L. (2019). Piezoelectric energy harvesting from human walking using a two-stage amplification mechanism. Energy.

[20] Asano S., Nishimura S., Ikeda Y., Nishimura S., Ikeda Y., Morita T., Hosaka H. (2020). Energy harvester for safety shoes using parallel piezoelectric links. Sens. Actuators A Phys..

[21] Wang S., Miao G., Zhou S., Yang Z., Yurchenko D. (2022). A novel electromagnetic energy harvester based on the bending of the sole. Appl. Energy.

[22] Wang Z., Wu X., Zhang Y., Liu Y., Liu Y., Cao W., Chen C. (2020). A New Portable Energy Harvesting Device Mounted on Shoes: Performance and Impact on Wearer. Energies.

[23] Luo A., Zhang Y., Dai X., Wang Y., Xu W., Lu Y., Wang M., Fan K., Wang F. (2020). An inertial rotary energy harvester for vibrations at ultra-low frequency with high energy conversion efficiency. Appl. Energy.

[24] Deng F., Cai Y., Fan X., Gui P., Chen J. (2019). Pressure-type generator for harvesting mechanical energy from human gait. Energy.

